# A latent profile analysis of emotional expression among patients with unintentional injuries

**DOI:** 10.1186/s12888-022-04390-4

**Published:** 2022-11-23

**Authors:** Xinlu Wang, Qiongyao Tu, Dongdong Huang, Pinpin Jin, Xue Cai, Haifeng Zhao, Zhongqiu Lu, Chaoqun Dong

**Affiliations:** 1 School of Nursing, Wenzhou Medical University, Wenzhou, Zhejiang China; 2 Ningbo City First Hospital, Ningbo, China; 3 Emergency Intensive Care Unit, Emergency Department, The First Affiliated Hospital of Wenzhou Medical University, Wenzhou, Zhejiang China

**Keywords:** Emotional expression, Unintentional injury, Latent profile analysis, Cognitive processing, Posttraumatic growth

## Abstract

**Background:**

Emotional expression has been suggested to affect the well-being of individuals with unintentional injuries. However, few studies have investigated it as a heterogeneous phenomenon. The purpose of this study was to characterize the patterns of emotional expression among patients with unintentional injuries using latent profile analysis, and to examine the relationship among these latent profiles and cognitive processing, posttraumatic growth, and posttraumatic stress disorder.

**Methods:**

A cross-sectional study was carried out at two general hospitals in Wenzhou, China. In total, 352 patients with unintentional injuries completed the socio-demographic questionnaire, Berkeley Expressivity Questionnaire, Ambivalence Over Emotional Expression Questionnaire, Event-Related Rumination Inventory, the Posttraumatic Growth Inventory, and PTSD Checklist-Civilian Version.

**Results:**

Three unique profiles were identified: high emotional expressivity (*n* = 238, 67.6%), moderate emotional expressivity (*n* = 45, 12.8%), and low emotional expressivity (*n* = 69, 19.6%). The ANOVA and chi-square tests demonstrated significant differences among the three groups concerning deliberate rumination and posttraumatic growth. Multinomial logistic regression analysis indicated that monthly income and time since injury significantly predicted profile membership.

**Conclusions:**

Most patients showed high emotional expressivity after an unintentional injury. Emotional expression profiles were associated with deliberate rumination and posttraumatic growth. Emotional expression interventions tailored for different profiles are warranted after an unintentional injury.

## Background

Unintentional injury is one of the leading causes of death and long-term disabilities worldwide, constituting a serious public health problem [1]. It has severe and continuous effects on survivors, including functional impairment, psychological suffering, and decreased quality of life [2]. Posttraumatic stress disorder (PTSD), depression, and other anxiety disorders are commonly reported among patients with unintentional injuries [3]. Nonetheless, some patients can experience positive psychological changes in the aftermath of unintentional injuries, even in the short-term [4, 5], which is a phenomenon defined as posttraumatic growth (PTG) [6]. Furthermore, despite PTG and PTSD being two opposing psychological outcomes, they have been shown to coexist in survivors of traumatic experiences [7, 8]. Although the influence mechanisms of PTG and PTSD are believed to be different [9], the underlying mechanisms remain unknown.

The PTG model proposes that emotional expression is one of the many potential factors that may affect the well-being of individuals who experience traumatic events [10]. Several studies have found that emotional expression is a facilitator of PTG [11, 12], and that a lower level of emotional expression is related to PTSD [13]. The PTG model also asserts that rumination, also expressed in a more neutral term as “cognitive processing,” is a potential mechanism that influences the relationship between emotional expression and psychological outcomes [10, 14]. It has been reported that intrusive rumination (uncontrollable recall of trauma-related cues) might contribute to PTSD, while deliberate rumination (active efforts to think about or reexamine traumatic events) could assist in the process of PTG [7]. Emotional expression can affect individuals’ cognitive processing by redirecting intrusive rumination into deliberate rumination and thus promote PTG [15, 16]. Meanwhile, emotional expression may significantly decrease PTSD symptoms through the mediating role of intrusive rumination [17]. Although emotional expression is believed to be one of the coping strategies to deal with traumatic events [18], some people may not be naturally inclined to express their emotions, which suggests that emotional expression-based interventions may not be suitable for individuals who are unwilling to express themselves [19]. Therefore, it is imperative to examine how the tendency to express emotions or the degree of emotional expressivity influences cognitive processing and psychological health in the aftermath of an unintentional injury.

Gross and John (1995) conceptualized emotional expressivity as the verbal and nonverbal behavioral adjustments resulting from emotional experiences. They also developed the Berkeley Expressivity Questionnaire (BEQ) to capture the multifactorial structure of emotional expression, including both the outward displays of positively/negatively valenced emotional expression (emotional expressive behavior) and the intensity of the internal emotional experience (emotional response tendency). However, other scholars proposed that the positive expressivity, negative expressivity, and strength of impulse facet subscales of the BEQ may be too broad to measure emotional expressivity and that cultural differences might strongly influence it [20]. Chinese cultures have a social convention related to the repression of emotions to avoid damaging social harmony [21]. A study that validated the BEQ in China revealed five domains of emotional expressivity: positive expressivity, negative expressivity, negative inhibition, positive impulse strength, and negative impulse strength [22]. Moreover, people’s internal struggles between the desire to express emotion and the fear of doing so, namely ambivalence over the expression of emotion (AEE), are also viewed as a specific facet of emotional expressivity [23].

Theoretically, the more expressive the individuals’ report, the more likely they experienced these emotions with a higher intensity [24]. However, the individual experience of a certain emotion does not necessarily guarantee its emotional expression, meaning that the forms by which people express their emotions vary. Specifically, researchers have shown that some people express their emotions regularly and frequently, regardless of emotion valence; others often inhibit their emotions [25, 26]; and while some emotions are allowed to be freely expressed, others are suppressed [27]. However, previous studies documenting emotional expression have mainly explored the different domains of emotional expression separately [28, 29] or merely focused on the broad concept of emotional expression [18, 30]. These variable-centered approaches neglect the fact that the expression of emotion is a heterogeneous phenomenon, entailing that investigating a single aspect is not enough to provide insight into the distinct patterns of emotional expression behavior in patients with unintentional injuries. Hence, a person-centered approach, such as latent profile analysis (LPA), may provide an alternative. LPA has been widely applied in psychology and humanities research to identify types of people who have divergent personal attribute profiles [31]. Such an approach can help us better understand the demographic differences related to emotional expression profiles and facilitate the development of targeted intervention strategies for patients with unintentional injuries. To the best of our knowledge, no recent study has updated the available information on the heterogeneity of the emotional expression subgroups in patients with unintentional injuries.

Several researchers have found that emotional expression reduces distress and that any barriers to it may inhibit the cognitive processing of stressful events [17, 32]. Individuals who are unable to express their emotions or do not want to do so have an increased risk of experiencing symptoms of PTSD [33]. Moreover, the desire to express, coupled with the lack of emotional expression, could result in intrusive thoughts [25]. However, the empirical findings on the beneficial effects of emotional expression are somewhat mixed. While some studies found that emotional expression may have health benefits by reducing negative emotions [34] and promoting PTG [35], others did not support the role of emotional expression in managing stress and improving cognitive processing [19, 36]. Furthermore, the relationship among patterns of emotional expression, cognitive processing, and posttraumatic outcomes is not well characterized.

Accordingly, this study aimed to: (1) identify homogenous groups of patients with unintentional injuries based on the latent profile of their emotional expression; (2) examine the socio-demographic correlates of these profiles; and (3) investigate the relationship among these latent profiles and cognitive processing, PTG, and PTSD.

## Methods

### Participants

An exploratory cross-sectional study was conducted in Wenzhou, China, from August 2018 to January 2019, and a convenience method was used to recruit participants from two general hospitals. As previous research suggested a minimum sample size of 300–500 for LPA studies [37], a total of 390 patients with unintentional injuries were involved in this study. The eligibility criteria for participants were: (a) having experienced an unintentional injury (e.g., traffic accidents, work-related injury, etc.) and requiring hospital admission for the treatment of physical injuries or functional impairment, (b) being aged 18–65 years old, and (c) having a clear mind and being willing to participate in the research. The exclusion criteria consisted of having a diagnosis of psychiatric illnesses or cognitive impairments provided by clinicians.

### Procedures

The aims and other details of the study were explained to the participants. All patients signed a written informed consent form. The patients were subsequently asked to complete a paper-and-pencil questionnaire. For those who could not read or write, the researcher read out each item and completed the questionnaires according to the person’s responses. This study was approved by the Institutional Ethics Committee of the Medical University (No. 2018043), and it followed the principles set forth in the Declaration of Helsinki [38].

### Measures

#### Socio-demographic questionnaire

The self-designed questionnaire was used to obtain socio-demographic variables including age, ethnicity, gender, marital status, monthly income, educational level, religion, employment condition, cause of injury, and time since injury. The questionnaire was filled out by patients independently and verified based on electronic medical records. With regards to the time since injury, patients were asked to fill out the duration from the first day they were injuried to the day they completed the questionnaire. We also collected data about the severity of patients’ injuries by using the Injury Severity Score (ISS). The ISS [39] was based on the Abbreviated Injury Scale (AIS) and used to describe the subjective severity of multiple injuries. In this scale, each injury is categorized into six body regions and assigned an AIS score, where the AIS is a 6-point ordinal scale (from 1, mild; to 6, fatal). The ISS is calculated by squaring and adding the scores of the three most severely wounded bodily regions, and it ranges from 0 to 75. An ISS value of less than 9 implies mild injuries, and a value of 9 or greater implies moderate to severe injuries [40].

#### Berkeley Expressivity Questionnaire (BEQ)

The Chinese version of the BEQ [22] was employed to measure emotional expressivity characteristics. It is a 16-item self-report scale (e.g., “I've learned it is better to suppress my anger than to show it.”), including five dimensions: positive expressivity, negative expressivity, negative inhibition, positive impulse strength, and negative impulse strength. Each item is rated on a 7-point Likert scale (from 1 = strongly disagree to 7 = strongly agree). Scores were calculated for each dimension, with higher scores suggesting greater strength of emotional response tendencies and the extent to which emotions were expressed. In this study, the Cronbach’s α for this scale was 0.71.

#### Ambivalence Over Emotional Expression Questionnaire (AEQ)

The Chinese version of AEQ [21] was used to assess an individual’s feelings of ambivalence over emotional expression. It is a single-dimension self-report measure with 24 items (e.g., “I worry that if I express negative emotions such as fear and anger, other people will not approve of me.”). Each item is rated on a 5-point scale (from 1 = completely inconsistent to 5 = completely consistent). The overall score ranges from 24 to 120, with higher values suggesting more psychological contradictions in the individual’s emotional expression. In this study, the Cronbach’s α for AEQ was 0.8.

#### Event-Related Rumination Inventory (ERRI)

The Chinese version of ERRI [41] was used to measure cognitive processing in the aftermath of unintentional injury and included 20 items (e.g., “I thought about the event when I did not mean to.”). It is a scale with two dimensions: intrusive rumination and deliberate rumination. This self-report questionnaire asked the participants to rate their thoughts that occurred in the previous two weeks on a 4-point Likert scale (from 0 = not at all to 3 = often). In this study, the Cronbach’s α for ERRI was 0.81.

#### Posttraumatic Growth Inventory (PTGI)

The Chinese version of PTGI [42], which was translated and modified based on the PTGI developed by Tedeschi and Calhoun [43], was used to evaluate psychological growth after the traumatic event, and included 20 items (e.g., “I can better appreciate each day.”). It has four dimensions: relating to others, new possibilities, personal strength, and application of life. Participants responded on a 6-point Likert scale (from 0 = strongly disagree to 5 = strongly agree). The total scores range from 0 to 100, and higher scores indicate greater PTG acquisition. In this study, the Cronbach’s α for PTGI-C was 0.85.

#### PTSD Checklist-Civilian Version (PCL-C)

The Chinese version of PCL-C [44] was used to assess posttraumatic stress symptoms after the traumatic events and included 17 items (e.g., “Having difficulty concentrating?”). It is a self-report scale with three dimensions: re-experience, avoidance, and hyperarousal. Each item is rated on a 5-point scale (from 1 = not at all to 5 = extremely), with total scores ranging from 17 to 85. A cut-off score of 38 was used to identify patients with PTSD symptoms [45]. In this study, the Cronbach’s α for PCL-C was 0.85.

### Data analysis

The data were analyzed using Mplus version 8.3 [46] and IBM SPSS Statistics version 25.0 [47]. Since our data were collected through the self-report method and from the same source, Harman’s single-factor test was conducted before data analysis, using exploratory factor analysis to detect common method bias. The results revealed that there were 25 factors with eigenvalues greater than 1, explaining 67.6% of the variance. The variance explained by the first factor was 14.3%, which is far less than the critical value of 40% [48]. Hence, there is no significant common method bias in this study.

Data analysis consisted of three parts. First, descriptive statistics were conducted for all variables to understand the basic characteristics of the patients. Continuous variables were presented in mean ± SD, and categorical variables were presented in frequencies and proportions. Second, LPA was performed to identify the emotional expression profiles of the 352 participants based on six continuous variables (positive expressivity, negative expressivity, negative inhibition, positive impulse strength, negative impulse strength, and ambivalence over emotional expression). To ensure comparability among items, we averaged the total scores of each subscale of the BEQ. We explored models that would identify one to five classes, and used the following fit indicators to determine the optimal number of latent profiles: Akaike information criterion (AIC), Bayesian information criterion (BIC), and adjusted Bayesian information criterion (aBIC) were employed to compare model, with lower AIC, BIC, and aBIC values indicating a better model fit; the Lo-Mendell-Rubin (LMR) and bootstrap likelihood ratio test (BLRT) were examined to identify whether a k-class model fit better than a model with k-1 classes, and a significant p*-*value indicated that the k class was better; the entropy was assessed to identify each model’s classification precision with greater values implying more precise categorization (ideally above 0.80) [49]. Third, a detailed description of each profile was provided after the groups had been established. We used a one-way ANOVA and chi-squared test to analyze continuous variables and categorical variables, respectively, to compare differences in socio-demographic characteristics among the subgroups. Multivariate multinomial logistic regression was then performed to examine the socio-demographic variables’ influence on each profile. Next, the association of profile membership with cognitive processing, PTG, and PTSD was assessed using an ANOVA or chi-squared test. Significance was set at a *p*-value < 0.05 for all analyses.

## Results

### Participant characteristics

Due to incomplete answers, data from 38 of the 390 eligible patients who agreed to participate were excluded, which left us with a valid sample of 352 participants (90.3%), including 284 male (80.7%) and 68 female participants (19.3%). The sample’s average age was 40.54 ± 11.30 years (range, 18–65). The majority of participants had an education level of middle school or below (75.6%, *n* = 266), were employed (83.8%, *n* = 295), were religiously unaffiliated (66.8%, *n* = 235), and were married (81.3%, *n* = 286). A total of 147 (41.8%) patients were reported to be injured for 1–7 days, 119 (33.8%) for 8–14 days, 56 (15.9%) for 15–30 days, and 30 (8.5%) for more than 30 days. Regarding the monthly income, 306 (86.9%) of the patients reported a monthly income of > 3000 renminbi (RMB, Chinese currency). In terms of the cause of injury, 127 (36.1%) were injured in their work, 102 (29.0%) were injured in a traffic accident, and 123 (34.9%) by other accidents. A total of 239 (67.9%) patients had an injury severity score (ISS) of less than 9.

### Latent profile analysis

**Fig. 1:**
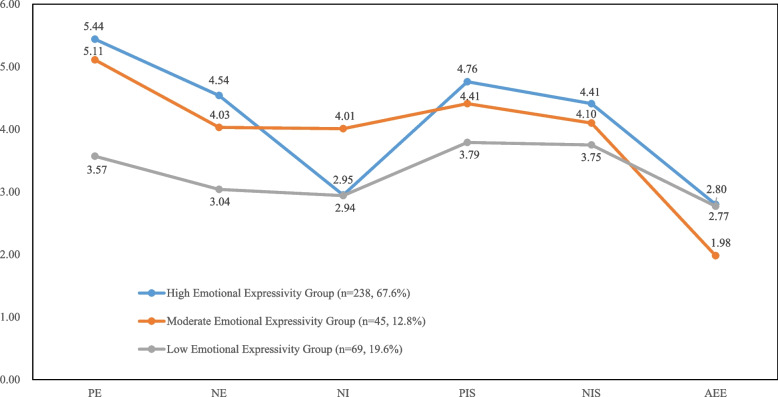
Latent Profile indicators mean values for the three-profile solution. Note. PE = Positive Expressivity. NE = Negative Expressivity. NI = Negative Inhibition. PIS = Positive Impulse Strength. NIS = Negative Impulse Strength. AEE = Ambivalence over Emotional Expression

Table 1 shows the model fit indices for the one-class to the five-class solutions. The AIC, BIC, and aBIC generally decreased as the number of estimated profiles increased, while the entropy stayed consistently above 0.80. According to LMR, the four-class solution did not enhance model fit considerably compared with the three-class solution (*p* = 0.33). Based on model fit tests and the goal of parsimony, the three-class solution was identified as the best description of latent emotional expressivity profiles. The latent profile memberships showed significant differences in the means of the six indicator variables (Table 2), and their characteristics are summarized in Fig. 1. Class 1 (*n* = 238, 67.6%), the *high emotional expressivity group*, was characterized by the highest emotional expressivity and intensity, the lowest inhibition of negative emotion expression, and the relatively high ambivalence over emotional expression. Class 2 (*n* = 45, 12.8%), the *moderate emotional expressivity group*, was distinguished by a moderate level of emotional expressivity and intensity, the highest inhibition of negative emotion expression, and the lowest level of ambivalence over emotional expression. Class 3 (*n* = 69, 19.6%), the *low emotional expressivity group*, was characterized by the lowest level in all emotional expressivity subscales, except for the relatively high ambivalence over emotional expression.

**Table 1:** Fit Statistics for the latent profile analysis

	AIC	BIC	aBIC	Entropy	LMRT	BLRT
					*p*-value	*p*-value
One-class	5164.46	5210.82	5172.76			
Two-class	4969.25	5042.66	4982.39	0.84	< 0.001	< 0.001
**Three-class**	**4920.16**	**5020.61**	**4938.13**	**0.82**	**0.03**	** < 0.001**
Four-class	4888.58	5016.08	4911.39	0.83	0.33	< 0.001
Five-class	4868.16	5022.71	4895.81	0.85	0.73	< 0.001

Note*.* Boldface indicates the selected model

**Table 2:** Descriptive statistics for indicator variables that constituted the three profiles

Variable	High Emotional Expressivity Group	Moderate Emotional Expressivity Group	Low Emotional Expressivity Group	F
	M (SD)	M (SD)	M (SD)	
PE	5.44 (0.63)	5.11 (0.58)	3.57 (0.79)	216.20***
NE	4.54 (0.78)	4.03 (0.71)	3.04 (0.68)	108.00***
NI	2.95 (0.87)	4.01 (0.64)	2.94 (0.86)	31.74***
PIS	4.76 (0.82)	4.41 (0.85)	3.79 (0.99)	34.35***
NIS	4.41(0.82)	4.10 (0.89)	3.75 (1.09)	15.17***
AEE	2.80 (0.29)	1.98 (0.26)	2.77 (0.35)	142.84***

Note*. PE* Positive Expressivity, *NE* Negative Expressivity, *NI* Negative Inhibition, *PIS* Positive Impulse Strength, *NIS* Negative Impulse Strength, *AEE* Ambivalence over Emotional Expression

^***^ *p* < 0.001

### Predictor of latent profile membership

The chi-squared test revealed significant differences between the three profiles regarding employment conditions ( χ^2^ = 9.21, *p* = 0.01), monthly income ( χ^2^ = 24.78, *p* < 0.001), and time since injury ( χ^2^ = 18.04, *p* = 0.01; Table 3). Using the *high emotional expressivity* or *low emotional expressivity* as the reference group, a multivariate multinomial logistic regression was conducted to investigate the socio-demographic predictors of profile membership (Table 4). When compared to those with a monthly income of ≤ 3000 RMB, patients with a monthly income of 3001–5000 RMB and 5001–8000 RMB had lower odds of being in the *moderate emotional expressivity group* than in the *high emotional expressivity group* (OR: 0.21, CI: 0.07—0.65 and OR: 0.22, CI: 0.07—0.68, respectively) and the *low emotional expressivity group* (OR: 0.24, CI: 0.06—0.89 and OR: 0.20, CI: 0.05—0.77, respectively), and patients with a monthly income of > 8000 RMB had lower odds of being in the *moderate emotional expressivity group* than in the *high emotional expressivity group* (OR: 0.23, CI: 0.07—0.73)*.* Patients who were injured for one week (compared to being injured for more than one month) were less likely to belong to the *low emotional expressivity group* than to the *high emotional expressivity group* (OR: 0.31, CI: 0.12—0.81).

**Table 3:** Descriptive statistics in the full sample and each latent profile

Variables	Total sample	High Emotional Expressivity Group	Moderate Emotional Expressivity Group	Low Emotional Expressivity Group	F/χ^2^	p
Age M (SD)	40.54 (11.30)	40.39 (10.92)	42.78 (11.33)	39.62 (12.48)	1.13	0.32
Ethnicity						
Han nationality	330 (93.8%)	223 (93.7%)	43 (95.6%)	64 (92.8%)	0.37	0.83
Minority	22 (6.3%)	15 (6.3%)	2 (4.4%)	5 (7.3%)		
Gender						
Male	284 (80.7%)	194 (82.5%)	33 (73.3%)	57 (82.6%)	1.83	0.40
Female	68 (19.3%)	44 (18.5%)	12 (17.4%)	12 (17.4%)		
Marriage status						
Married	286 (81.3%)	200 (84.0%)	33 (73.3%)	53 (76.8%)	3.95	0.14
Single/Divorced/widowed	66 (18.8%)	38 (16.0%)	12 (26.7%)	16 (23.2%)		
Educational level						
Middle school/lower	266 (75.6%)	173 (72.7%)	37 (82.2%)	56 (81.2%)	3.32	0.19
High school/higher	86 (24.4%)	65 (27.3%)	8 (17.8%)	13 (18.8%)		
Religion						
No	235 (66.8%)	156 (65.6%)	31 (68.9%)	48 (69.6%)	0.50	0.78
Yes	117 (33.2%)	82 (34.5%)	14 (31.1%)	21 (30.4%)		
Employment Condition						
Employed	295 (83.8%)	207 (87.0%)	31 (68.9%)	57 (82.6%)	9.21	0.01
Not Employed	57 (16.2%)	31 (13.0%)	14 (31.1%)	12 (17.4%)		
Monthly income (RMB)						
≤ 3000	46 (13.1%)	21 (8.8%)	16 (35.6%)	9 (13.0%)	24.78	< 0.001
3001–5000	93 (26.4%)	65 (27.3%)	9 (20.0%)	19 (27.5%)		
5001–8000	100 (28.4%)	69 (29.0%)	9 (20.0%)	22 (31.9%)		
> 8000	113 (32.1%)	83 (34.9%)	11 (24.4%)	19 (27.5%)		
ISS score						
< 9	239 (67.9%)	164 (68.9%)	28 (62.2%)	47 (68.1%)	0.78	0.68
≥ 9	113 (32.1%)	74 (31.1%)	17 (37.8%)	22 (31.9%)		
Cause of injury						
Work-related injury	127 (36.1%)	91 (38.2%)	13 (28.9%)	23 (33.3%)	2.35	0.67
Traffic accident injury	102 (29.0%)	67 (28.2%)	16 (35.6%)	19 (27.5%)		
Other	123 (34.9%)	80 (33.6%)	16 (35.6%)	27 (39.1%)		
Time since injury (days)						
1–7	147 (41.8%)	114 (47.9%)	11 (24.4%)	22 (31.9%)	18.04	0.01
8–14	119 (33.8%)	76 (31.9%)	22 (48.9%)	21 (30.4%)		
15–30	56 (15.9%)	33 (13.9%)	7 (15.6%)	16 (23.2%)		
> 30	30 (8.5%)	15 (6.3%)	5 (11.1%)	10 (14.5%)

**Table 4:** Multivariate multinomial logistic regression results predicting profile membership

Variables	Moderate Emotional Expressivity Group vs High Emotional Expressivity Group		Low Emotional Expressivity Group vs High Emotional Expressivity Group		Moderate Emotional Expressivity Group vs Low Emotional Expressivity Group	
	OR (95%CI)	p	OR (95%CI)	p	OR (95%CI)	p
Age M (SD)	1.01 (0.52–1.93)	0.99	1.18 (0.71–1.98)	0.53	0.85 (0.41–1.79)	0.67
Ethnicity					Ref	
Han nationality	1.92 (0.38–9.85)	0.43	0.93 (0.30–2.93)	0.90	2.07 (0.34–12.44)	0.43
Minority	Ref		Ref		Ref	
Gender						
Male	1.08 (0.46–2.58)	0.86	1.36 (0.63–2.95)	0.43	0.79 (0.28–2.22)	0.66
Female	Ref		Ref		Ref	
Marriage status						
Married	0.50 (0.21–1.21)	0.12	0.53 (0.25–1.11)	0.09	0.95 (0.35–2.59)	0.92
Single/Divorced/widowed	Ref		Ref		Ref	
Educational level						
Middle school/lower	1.51 (0.61–3.74)	0.38	1.66 (0.81–3.41)	0.17	0.91 (0.31–2.64)	0.86
High school/higher	Ref		Ref		Ref	
Religion						
No	1.24 (0.58–2.67)	0.58	1.18 (0.63–2.19)	0.61	1.06 (0.43–2.57)	0.91
Yes	Ref				Ref	
Employment Condition						
Employed	0.80 (0.30–2.15)	0.66	0.94 (0.38–2.31)	0.89	0.85 (0.27–2.71)	0.79
Not Employed	Ref		Ref		Ref	
Monthly income (RMB)						
≤ 3000	Ref		Ref		Ref	
3001–5000	0.21 (0.07–0.65)	0.01	0.91 (0.31–2.68)	0.87	0.24 (0.06–0.89)	0.03
5001–8000	0.22 (0.07–0.68)	0.01	1.09 (0.37–3.18)	0.88	0.20 (0.05–0.77)	0.02
> 8000	0.23 (0.07–0.73)	0.01	0.85 (0.27–2.67)	0.78	0.27 (0.07–1.10)	0.07
ISS score						
< 9	0.85 (0.41–1.76)	0.67	1.10 (0.60–2.04)	0.75	0.77 (0.33–1.80)	0.55
≥ 9	Ref		Ref		Ref	
Cause of injury						
Work-related injury	1.21 (0.48–3.07)	0.69	0.68 (0.34–1.39)	0.29	1.77 (0.62–5.10)	0.29
Traffic accident injury	1.47 (0.61–3.51)	0.39	0.77 (0.37–1.60)	0.49	1.90 (0.69–5.21)	0.22
Other	Ref		Ref		Ref	
Time since injury (days)						
1–7	0.51 (0.14–1.85)	0.30	0.31 (0.12–0.81)	0.02	1.66 (0.40–6.79)	0.48
8–14	1.03 (0.31–3.43)	0.97	0.42 (0.16–1.10)	0.08	2.45 (0.66–9.12)	0.18
15–30	1.04 (0.26–4.17)	0.96	0.84 (0.30–2.38)	0.74	1.24 (0.28–5.45)	0.78
> 30	Ref		Ref		Ref	

Note. *OR* odds ratio, *CI* confidence interval

### Cognitive processing, PTG, and PTSD across the identified latent profiles

The one-way ANOVA (Table 5) showed a significant difference among latent profile groups for deliberate rumination (*F* = 13.79, *p* < 0.001), PTG total score (*F* = 3.04, *p* = 0.04), relating to others (*F* = 4.69, *p* = 0.01) and new possibilities (*F* = 3.67, *p* = 0.03). Patients in the *moderate emotional expressivity group* scored the lowest (M = 9.00, SD = 4.50) on deliberate rumination, and those in the *low emotional expressivity group* scored the lowest on PTG total score (M = 38.52, SD = 14.33), relating to others (M = 8.70, SD = 3.61) and new possibilities (M = 7.54, SD = 4.91). Furthermore, no direct relationship was identified between latent profiles and the occurrence of PTSD in the Chi-squared test (Table 6).

**Table 5:** Mean scores and standard deviations across latent profiles on Cognitive processing and PTG

Variables	High Emotional Expressivity Group M (SD)	Moderate Emotional Expressivity Group M (SD)	Low Emotional Expressivity Group M (SD)	F	p	Post hoc analysis
Cognitive processing						
Intrusive rumination	9.22 (6.42)	7.60 (5.38)	8.91 (5.97)	1.29	0.28	
Deliberate rumination	12.75 (4.48)	9.00 (4.50)	11.41 (4.67)	13.79	< 0.001	1 > 3 > 2
PTG						
Relating to others	10.08 (3.15)	9.80 (3.72)	8.70 (3.61)	4.69	0.01	1 > 3
New possibilities	8.49 (4.12)	9.76 (4.12)	7.54 (4.91)	3.67	0.03	1 > 3, 2 > 3
Personal strength	12.28 (4.30)	12.42 (3.79)	11.70 (5.45)	0.52	0.59	
Appreciation of life	11.79 (4.64)	11.69 (4.21)	10.59 (4.84)	1.81	0.17	
Total score	42.64 (12.87)	43.67 (12.95)	38.52 (14.33)	3.04	0.04	1 = 2 > 3

Note. 1 = High Emotional Expressivity Group; 2 = Moderate Emotional Expressivity Group; 3 = Low Emotional Expressivity Group

**Table 6:** Relationship between latent profiles and the prevalence of PTSD

	Total	High Emotional Expressivity Group	Moderate Emotional Expressivity Group	Low Emotional Expressivity Group	χ^2^	p
Yes	24 (6.8%)	20 (8.4%)	1 (2.2%)	3 (4.4%)	3.10	0.21
No	328 (93.2%)	218 (91.6%)	44 (97.8%)	66 (95.7%)		

## Discussion

Using the LPA technique, we grouped patients with unintentional injuries by their emotional expression and identified three distinct profiles: *high emotional expressivity group*, *moderate emotional expressivity group*, and *low emotional expressivity group*. The largest proportion of patients belonged to the *high emotional expressivity group*. Most of the patients in the aftermath of unintentional injuries experienced a strong intensity of inner emotions and could express their positive and negative emotions outwardly. This high emotional expressivity may reduce their tendency to suppress negative emotions, resulting in the lowest inhibition of negative expressivity in this group. Additionally, patients in this group may still feel ambivalent about expressing their feelings. This result supports the assertion that individuals may still experience emotional conflicts despite being highly expressive [50]. Unsurprisingly, patients in the first week after unintentional injuries were more likely to belong to the *high emotional expressivity group* since they were overwhelmed by diverse emotions immediately after their injuries [51]. Therefore, healthcare providers should provide a supportive environment for patients to share their emotions soon after the accident. The participants in this group also reported a higher level of deliberate rumination and PTG, which are in accordance with the PTG theory that expressing one’s emotions regarding the traumatic experience may make informational resources available by enhancing the cognitive processing of the experience [10]. Emotional expression facilitates the process wherein one goes from ruminating about emotional reactions to more goal-oriented thinking [52], which, in turn, may promote posttraumatic adjustment. Future research should focus on how emotional expression patterns interact with cognitive processing to influence psychological adjustment following an unintentional injury.

In contrast to the previous evidence showing that expressive inhibition is a possible mechanism in post-trauma affect dysregulation [53], our study found that the *moderate emotional expressivity group* with the highest inhibition of negative expressivity also reported a high level of PTG both in a whole and in its subscales of new possibility. This result might be because emotional suppression can also show the adaptational consequence in a culture where individuals are encouraged to distance themselves from negative emotional experiences [54]. Participants in the *moderate emotional expressivity group* also experienced the lowest level of ambivalence over emotional expression. This result corroborates prior research, which asserts that ambivalence over emotional expression is a crucial component in distinguishing a healthy style of emotional expression from an unhealthy style [55–57], regardless of whether the individual is behaviorally expressive. Surprisingly, patients with a higher monthly income (> 3000 RMB) had a greater chance of being both in the *high emotional expressivity group* and the *low emotional expressivity group*. Researchers have suggested that subjective socioeconomic status, rather than objective socioeconomic status, is a predictor of emotional expression [58]. As a result, more research is needed to confirm the link between socioeconomic level and emotional expression patterns.

Patients in the *low emotional expressivity group* stood out as the dysfunctional disclosure tendency, which was characterized by the lowest emotional expressivity and highest ambivalence over emotional expression. The participants in this group also reported the lowest PTG, thus concurring with the extensive evidence about emotional expression and PTG [18, 59]. Individuals with low emotional expressivity usually find it hard to develop and maintain close relationships with others [60], which may adversely affect the perceived PTG in terms of relating to others. People who are strongly ambivalent over emotional expression and coupled with low emotional expressivity have a low ability to identify and understand their distress and consequently do not invest in the positive reappraisal of the traumatic events [61], which accounts for the lowest level of new possibilities in this group. Hence, interventions aimed at enhancing emotional expressivity among patients with unintentional injuries seem to be of high clinical relevance for patients’ psychological growth. Moreover, we did not find any association between the participants’ emotional expression patterns and PTSD prevalence, which is in line with previous research conducted on a community sample [33] and veterans with PTSD [62]. As negative reactions to emotional disclosure will inhibit the tendency of people to express their emotions and increase internal conflicts about emotional expression [63], health caregivers need to provide supportive reactions to facilitate and encourage emotional expression, especially for those who are ambivalent in this regard.

Clinically, this study provided evidence for the implementation of emotional expression interventions to promote PTG in patients with unintentional injuries. Our findings may help healthcare professionals identify subgroups of patients who are at higher risk of low levels of PTG by their emotional expression patterns. Healthcare professionals should pay more attention to patients with low emotional expressivity and high ambivalence over emotional expression simultaneously. Interventions that do not increase patients’ fear of judgment from others, such as private writing-based emotional expression [64] and peer social support group interventions [65], may be effective in promoting their emotional disclosure and reducing their reluctance to express emotions. Considering patients’ high emotional expressivity in the first week after injuries, it is important for healthcare professionals to implement emotional expression interventions as soon as possible. However, as there are few emotional expression interventions designed for unintentionally injured patients in China, the development of culturally sensitive and effective interventions for this vulnerable population is imperative.

This study has several limitations that need to be acknowledged. First, we used a convenience sample of patients from a single region, so it is not representative of patients from other areas. Thus, results should be generalized with caution. Additionally, most participants were within one month after unintentional injuries and their PTSD symptom levels were fairly low, and these characteristics might have led to the underestimation of the association between emotional expression and PTSD. Second, the cross-sectional design limits our ability to draw causal conclusions, thus entailing that longitudinal research should be conducted to confirm the significance of emotional expression on posttraumatic outcomes. Third, as the sample size of the *moderate* and *low emotional expressivity group* was rather small, future research on similar topics should consider expanding the sample size. Fourth, we used only two questionnaires to capture the characteristics of emotional expression. Future studies should measure more aspects of emotional expression. Furthermore, as emotional expression involves a complex interplay between intrapersonal and interpersonal processes, additional studies to assess the social reactions toward emotional expression are needed to obtain a comprehensive understanding.

To our knowledge, this study is the first to explore the patterns of emotional expression of patients after an unintentional injury. We identified three distinct patterns of emotional expression and found that patients with low emotional expressivity exhibit the lowest level of PTG. Our findings can be used to guide future studies regarding the effects of emotional expression on posttraumatic adjustment. Moreover, our results underline the importance of comprehensively assessing emotional expressivity before the intervention. Demographic variation in emotional expression patterns suggests that targeted interventions are warranted to promote psychological adjustment after an unintentional injury.

## Data Availability

The authors confirm that the data supporting the findings of this study are available within the article.
